# *N*-Hexyl-3-(4-hy­droxy-3,5-dimeth­oxy­phen­yl)propanamide

**DOI:** 10.1107/S1600536812019022

**Published:** 2012-05-02

**Authors:** L. C. R. Andrade, J. A. Paixão, M. J. M. de Almeida, E. J. Tavares da Silva, F. M. Fernandes Roleira

**Affiliations:** a CEMDRX, Department of Physics, Faculty of Sciences and Technology, University of Coimbra, P-3004-516 Coimbra, Portugal; b Center for Pharmaceutical Studies, Pharmaceutical Chemistry Group, Faculty of Pharmacy, University of Coimbra, P-3000-548 Coimbra, Portugal

## Abstract

In the title compound, C_17_H_27_NO_4_, which is an hydro­sinapic acid derivative with increased lipophilicity conferred by an additional alkyl chain, the central and the hexyl linear chains contain slightly shorter bond lengths [C—N = 1.316 (2) Å; average linear chain C—C = 1.487 (6) Å] than reported average values [C*sp* ^2^—N = 1.334, C—C for CH_2_—CH_2_ = 1.524 and 1.513 Å for CH_2_—CH_3_]. The 4-hy­droxy-3,5-dimeth­oxy­phenyl plane [r.m.s. deviation 0.055 (12) Å] makes an angle of 59.89 (5)° with the central plane of the mol­ecule (composed of the N atom, the carbonyl group and the two methyl­ene C atoms linking the carbonyl group and the ring, [r.m.s. deviation 0.0026 (10) Å], which, in turn, makes an angle of 64.24 (13)° with the essentially planar hexyl chain [r.m.s. deviation 0.035 (18) Å]. The N—H group of the amide group is involved in a bifurcated hydrogen bond towards the hy­droxy and one of the meth­oxy O atoms of the 4-hy­droxy-3,5-dimeth­oxy­phenyl substituent of a neighbouring mol­ecule, forming a two-dimensional network in the (100) plane. In addition, the same hy­droxy group acts as a donor towards the carbonyl O atom of another neighbouring mol­ecule, forming chains running along the *b* axis.

## Related literature

For the dependence on their structural characteristics of the anti­cancer activity of phenolic acids and their derivatives, see: Gomes *et al.* (2003 ▶). For restrictions on protection of lipophilic systems due to the hydro­philic nature of mol­ecules in aqueous media, see: Gao & Hu (2010 ▶). For the synthesis, see: Roleira *et al.* (2010 ▶). For reference bond lengths, see: Allen *et al.* (1987 ▶).
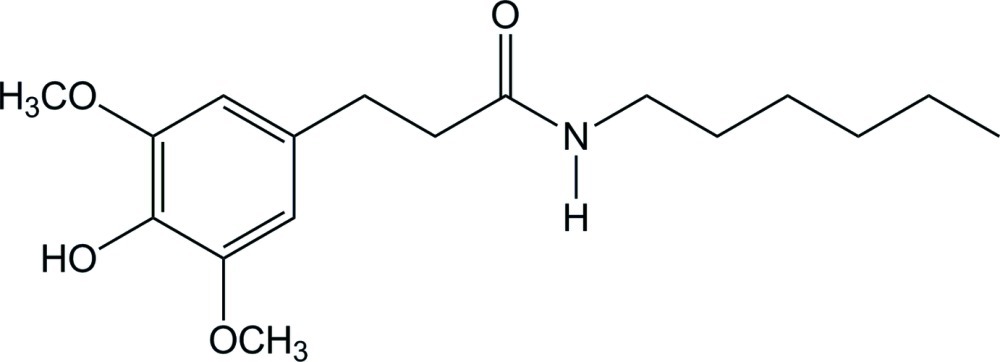


## Experimental

### 

#### Crystal data

C_17_H_27_NO_4_
*M* *_r_* = 309.40
Monoclinic, 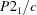

*a* = 19.1126 (5) Å
*b* = 8.4086 (2) Å
*c* = 11.0715 (3) Å
β = 91.5691 (15)°
*V* = 1778.64 (8) Å^3^
*Z* = 4
Mo *K*α radiation
μ = 0.08 mm^−1^
*T* = 293 K
0.34 × 0.26 × 0.19 mm

#### Data collection

Bruker APEX CCD diffractometer
Absorption correction: multi-scan (*SADABS*; Sheldrick, 2000 ▶) *T*_min_ = 0.856, *T*_max_ = 0.865
34604 measured reflections
4259 independent reflections
2478 reflections with *I* > 2σ(*I*)
*R*_int_ = 0.038

#### Refinement

*R*[*F* ^2^ > 2σ(*F* ^2^)] = 0.048
*wR*(*F* ^2^) = 0.172
*S* = 0.99
4255 reflections
204 parameters
H-atom parameters constrained
Δρ_max_ = 0.19 e Å^−3^
Δρ_min_ = −0.15 e Å^−3^

### 

Data collection: *SMART* (Bruker, 2006 ▶); cell refinement: *SAINT* (Bruker, 2006 ▶); data reduction: *SAINT*; program(s) used to solve structure: *SHELXS97* (Sheldrick, 2008 ▶); program(s) used to refine structure: *SHELXL97* (Sheldrick, 2008 ▶); molecular graphics: *PLATON* (Spek, 2009) ▶; software used to prepare material for publication: *SHELXL97*.

## Supplementary Material

Crystal structure: contains datablock(s) global, I. DOI: 10.1107/S1600536812019022/bt5889sup1.cif

Structure factors: contains datablock(s) I. DOI: 10.1107/S1600536812019022/bt5889Isup2.hkl

Supplementary material file. DOI: 10.1107/S1600536812019022/bt5889Isup3.cml

Additional supplementary materials:  crystallographic information; 3D view; checkCIF report

## Figures and Tables

**Table 1:** Hydrogen-bond geometry (Å, °)

*D*—H⋯*A*	*D*—H	H⋯*A*	*D*⋯*A*	*D*—H⋯*A*
N—H10⋯O4^i^	0.86	2.16	2.9655 (19)	155
N—H10⋯O5^i^	0.86	2.55	3.244 (2)	138
O4—H4⋯O9^ii^	0.82	1.84	2.6216 (17)	158

Symmetry codes: (i) 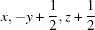; (ii) 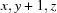.

**Fig. 1.**
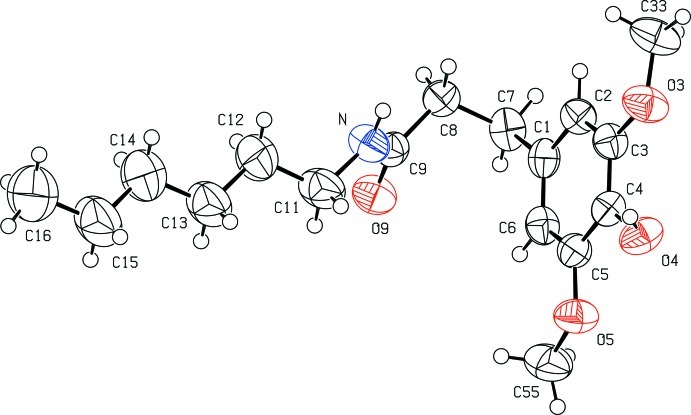
ORTEPII plot of the title compound. Displacement ellipsoids are drawn at the 50% level.

**Fig. 2.**
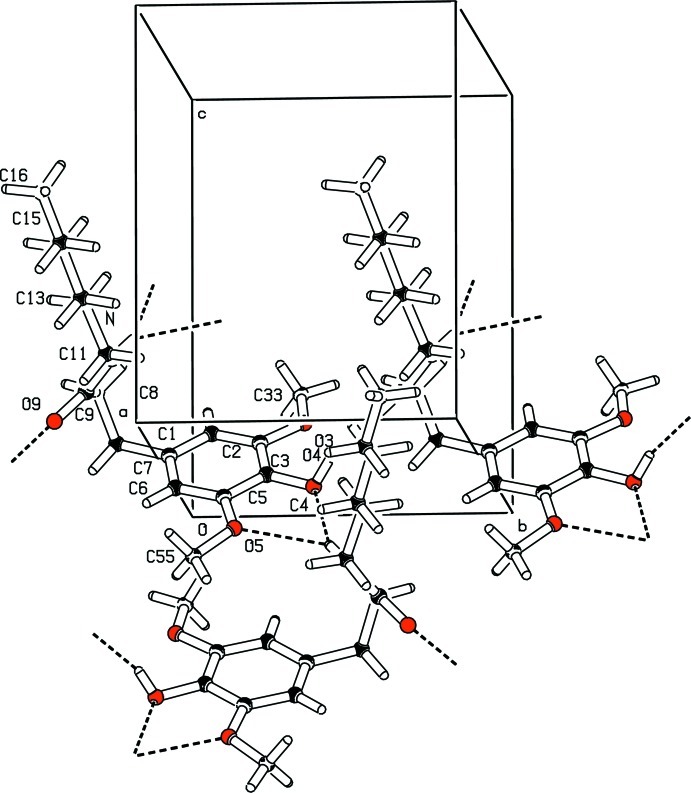
Diagram depicting the H-bond network.

## supplementary crystallographic information

### Comment

Hydroxycinnamic acids and derivatives are known to display relevant antioxidant properties as well as biological activity towards several tumor cells, with their growth-inhibitory potency being strongly dependent on their structural characteristics (Gomes *et al.*, 2003). Despite all the interesting biological effects of hydroxycinnamic acids and despite being dietary components, their bioavailability presents some limitations: although working well in aqueous media, their hydrophilic nature is usually a restriction for lipophilic systems protection (Gao & Hu, 2010). In order to develop new and more effective phenolic agents suitable for chemopreventive and/or chemotherapeutic purposes, hydrosinapic acid derivatives with increased lipophilicity conferred by an additional alkyl chain, were developed. For this, *N*-hexyl-3-(4-hydroxy-3,5-dimethoxyphenyl)propanamide was synthesized by reaction of the corresponding acid with hexylamine, in the presence of the coupling agent (benzotriazol-1-yloxy)tris(dimethylamino)phosphonium hexafluorophosphate (BOP) (Roleira *et al.*, 2010). Single crystal X-ray measurements evidence normal bond length values for the phenyl ring and its substituents. However the C*_sp_*^2^–N bond length in the molecule's central chain [1.316 (2) Å] is shorter than the reported average value of 1.334 Å (Allen *et al.*, 1987). Furthermore the average value of the five measured C*_sp_*^3^–C*_sp_*^3^ bond lengths of the hexyl chain [1.487 (6) Å] is also significantly shorter then the average reported values (1.524 for CH_2_–CH_2_ and 1.513 for CH_2_–CH_3_, Allen *et al.*, 1987). The molecule is characterized by an intramolecular C11–H11A···O9 pseudo-hydrogen bond within the central chain plane (deviation 0.0026 Å). The dihedral angle between this plane and the phenyl one (deviation 0.0545 Å) is 59.89 (5)°, being 64.24 (13)° the corresponding value between the central plane and the one of the hexyl chain (deviation 0.0349 Å). Cohesion of the structure is obtained through an extended newtork of H-bonds. The H atom of the amide group is involved in a bifurcated H-bond towards the hydroxy and one of the methoxy O atoms of the 4-hydroxy-3,5-dimethoxyphenyl substituent of a neignhbour molecule, forming a two dimensional network in the (100) plane. In addition, the same hydroxy group acts as a donnor towards the carbonyl O atom of another neighbour molecule forming chains running along the *b* axis.

### Experimental

The title amide was prepared from the 3-(4-hydroxy-3,5-dimethoxyphenyl)propanoic acid by dissolution of 5 mmol of the acid in 10 ml of DMF followed by the addition of triethylamine (0.7 ml, 5 mmol). The solution was cooled in an ice-water bath and 0.657 ml (5 mmol) of *N*-hexylamine were added followed by a solution of 2.21 g (5 mmol) of BOP in 10 ml of methylene chloride. The mixture was stirred at 273 K for 30 min and then at room temperature for 30 min. Methylene chloride was removed under reduced pressure and the solution was diluted with 150 ml of water and extracted with ethyl acetate (150 ml). The organic phase was washed successively with 1 N hydrochloride acid (3x100 ml), water (150 ml), 1*M* NaHCO_3_ (3x100 ml), and water (2x100 ml), dried over anhydrous magnesium sulfate, filtered and evaporated, affording a crude material which was purified by crystallization yielding the desired amide. Suitable crystals for X-ray analysis were grown from slow evaporation of ethyl acetate. Mp(ethyl acetate): 366–367 K; IR (ATR) υ_max_ cm^-1^: 3319 (N—H stretch), 1643 (C═O), 1125 (C–O).

### Refinement

All hydrogen atoms were placed at idealized positions and refined as riding on their parent atoms using *SHELXL97* defaults; the hydroxyl H atom was initialy positioned at the maximum of the difference electronic density around the parent O atom and refined using the HFIX 147 instruction.

Only 4255 out of 4259 independent reflections were used in the refinement because 4 low angle reflections were omitted due to overshadowing from the beam-stop.

### Crystal data

**Table:**

C_17_H_27_NO_4_	*D*_x_ = 1.157 Mg m^−^^3^
*M**_r_* = 309.40	Melting point: 366.5 K
Monoclinic, *P*2_1_/*c*	Mo *K*α radiation, λ = 0.71073 Å
*a* = 19.1126 (5) Å	Cell parameters from 7386 reflections
*b* = 8.4086 (2) Å	θ = 3.0–23.3°
*c* = 11.0715 (3) Å	µ = 0.08 mm^−^^1^
β = 91.5691 (15)°	*T* = 293 K
*V* = 1778.64 (8) Å^3^	Prism, colourless
*Z* = 4	0.34 × 0.26 × 0.19 mm
*F*(000) = 672	

### Data collection

**Table:**

Bruker APEX CCD diffractometer	4259 independent reflections
Radiation source: fine-focus sealed tube	2478 reflections with *I* > 2σ(*I*)
Graphite monochromator	*R*_int_ = 0.038
φ and ω scans	θ_max_ = 27.9°, θ_min_ = 2.7°
Absorption correction: multi-scan (*SADABS*; Sheldrick, 2000)	*h* = −25→25
*T*_min_ = 0.856, *T*_max_ = 0.865	*k* = −11→9
34604 measured reflections	*l* = −12→14

### Refinement

**Table:**

Refinement on *F*^2^	Secondary atom site location: difference Fourier map
Least-squares matrix: full	Hydrogen site location: inferred from neighbouring sites
*R*[*F*^2^ > 2σ(*F*^2^)] = 0.048	H-atom parameters constrained
*wR*(*F*^2^) = 0.172	*w* = 1/[σ^2^(*F*_o_^2^) + (0.094*P*)^2^ + 0.1703*P*] where *P* = (*F*_o_^2^ + 2*F*_c_^2^)/3
*S* = 0.99	(Δ/σ)_max_ < 0.001
4255 reflections	Δρ_max_ = 0.19 e Å^−^^3^
204 parameters	Δρ_min_ = −0.15 e Å^−^^3^
0 restraints	Extinction correction: *SHELXL97* (Sheldrick, 2008), Fc^*^=kFc[1+0.001xFc^2^λ^3^/sin(2θ)]^-1/4^
Primary atom site location: structure-invariant direct methods	Extinction coefficient: 0.014 (3)

### Special details

**Table:**

Geometry. All e.s.d.'s (except the e.s.d. in the dihedral angle between two l.s. planes) are estimated using the full covariance matrix. The cell e.s.d.'s are taken into account individually in the estimation of e.s.d.'s in distances, angles and torsion angles; correlations between e.s.d.'s in cell parameters are only used when they are defined by crystal symmetry. An approximate (isotropic) treatment of cell e.s.d.'s is used for estimating e.s.d.'s involving l.s. planes.
Refinement. Refinement of *F*^2^ against ALL reflections. The weighted *R*-factor *wR* and goodness of fit *S* are based on *F*^2^, conventional *R*-factors *R* are based on *F*, with *F* set to zero for negative *F*^2^. The threshold expression of *F*^2^ > σ(*F*^2^) is used only for calculating *R*-factors(gt) *etc*. and is not relevant to the choice of reflections for refinement. *R*-factors based on *F*^2^ are statistically about twice as large as those based on *F*, and *R*- factors based on ALL data will be even larger.

### Fractional atomic coordinates and isotropic or equivalent isotropic displacement parameters (Å^2^)

**Table:**

	*x*	*y*	*z*	*U*_iso_*/*U*_eq_	
N	0.75497 (7)	−0.11966 (19)	0.21012 (13)	0.0636 (4)	
H10	0.7363	−0.0564	0.2610	0.076*	
O3	0.58084 (7)	0.45097 (14)	0.08943 (12)	0.0698 (4)	
O4	0.67504 (7)	0.49686 (13)	−0.08385 (10)	0.0651 (4)	
H4	0.6877	0.5478	−0.0240	0.098*	
O5	0.73329 (7)	0.25857 (15)	−0.19467 (11)	0.0676 (4)	
O9	0.73578 (7)	−0.30205 (15)	0.06653 (12)	0.0689 (4)	
C1	0.61840 (9)	0.03560 (18)	0.00631 (14)	0.0507 (4)	
C2	0.58855 (9)	0.16335 (19)	0.06493 (15)	0.0523 (4)	
H2	0.5555	0.1454	0.1236	0.063*	
C3	0.60757 (9)	0.31751 (18)	0.03669 (15)	0.0511 (4)	
C4	0.65672 (9)	0.34669 (18)	−0.05009 (14)	0.0485 (4)	
C5	0.68601 (9)	0.21800 (19)	−0.10955 (14)	0.0510 (4)	
C6	0.66706 (9)	0.06345 (19)	−0.08136 (14)	0.0528 (4)	
H6	0.6871	−0.0216	−0.1215	0.063*	
C7	0.59855 (9)	−0.13192 (19)	0.04171 (16)	0.0585 (5)	
H7A	0.6098	−0.2039	−0.0234	0.070*	
H7B	0.5484	−0.1368	0.0525	0.070*	
C8	0.63594 (9)	−0.18697 (19)	0.15739 (15)	0.0549 (4)	
H8A	0.6284	−0.1097	0.2208	0.066*	
H8B	0.6161	−0.2874	0.1826	0.066*	
C9	0.71300 (9)	−0.20718 (18)	0.14130 (15)	0.0501 (4)	
C11	0.83047 (10)	−0.1231 (3)	0.20557 (19)	0.0850 (7)	
H11A	0.8472	−0.0163	0.1900	0.102*	
H11B	0.8438	−0.1897	0.1385	0.102*	
C12	0.86543 (12)	−0.1833 (3)	0.3176 (2)	0.0894 (7)	
H12A	0.8501	−0.2916	0.3316	0.107*	
H12B	0.8508	−0.1192	0.3852	0.107*	
C13	0.94383 (12)	−0.1808 (4)	0.3140 (2)	0.0967 (8)	
H13A	0.9580	−0.2479	0.2477	0.116*	
H13B	0.9585	−0.0731	0.2959	0.116*	
C14	0.98230 (14)	−0.2337 (4)	0.4256 (3)	0.1093 (9)	
H14A	0.9668	−0.1695	0.4927	0.131*	
H14B	0.9694	−0.3430	0.4420	0.131*	
C15	1.05973 (14)	−0.2242 (4)	0.4222 (3)	0.1218 (11)	
H15A	1.0751	−0.2876	0.3547	0.146*	
H15B	1.0726	−0.1148	0.4064	0.146*	
C16	1.09856 (17)	−0.2777 (5)	0.5325 (3)	0.1430 (13)	
H16A	1.0904	−0.3891	0.5448	0.215*	
H16B	1.0828	−0.2191	0.6009	0.215*	
H16C	1.1477	−0.2597	0.5232	0.215*	
C33	0.52544 (11)	0.4316 (2)	0.1699 (2)	0.0792 (6)	
H33A	0.5415	0.3707	0.2386	0.119*	
H33B	0.4874	0.3767	0.1296	0.119*	
H33C	0.5098	0.5340	0.1962	0.119*	
C55	0.76020 (12)	0.1358 (3)	−0.26704 (19)	0.0827 (6)	
H55A	0.7222	0.0765	−0.3033	0.124*	
H55B	0.7888	0.0663	−0.2178	0.124*	
H55C	0.7879	0.1812	−0.3293	0.124*	

### Atomic displacement parameters (Å^2^)

**Table:**

	*U*^11^	*U*^22^	*U*^33^	*U*^12^	*U*^13^	*U*^23^
N	0.0518 (10)	0.0748 (10)	0.0642 (9)	−0.0007 (7)	0.0016 (7)	−0.0170 (8)
O3	0.0784 (9)	0.0481 (7)	0.0842 (9)	0.0003 (6)	0.0272 (7)	−0.0118 (6)
O4	0.0938 (10)	0.0483 (7)	0.0537 (7)	−0.0083 (6)	0.0098 (6)	0.0001 (5)
O5	0.0770 (9)	0.0656 (8)	0.0611 (7)	−0.0018 (6)	0.0200 (6)	−0.0086 (6)
O9	0.0729 (9)	0.0600 (8)	0.0742 (8)	−0.0011 (6)	0.0102 (7)	−0.0160 (6)
C1	0.0504 (10)	0.0457 (8)	0.0552 (9)	−0.0015 (7)	−0.0134 (7)	−0.0021 (7)
C2	0.0495 (10)	0.0502 (9)	0.0573 (9)	−0.0036 (7)	0.0020 (8)	−0.0012 (8)
C3	0.0548 (10)	0.0442 (9)	0.0542 (9)	0.0016 (7)	0.0003 (7)	−0.0063 (7)
C4	0.0572 (10)	0.0426 (8)	0.0456 (8)	−0.0020 (7)	−0.0018 (7)	−0.0009 (7)
C5	0.0501 (10)	0.0564 (9)	0.0465 (8)	0.0004 (7)	−0.0017 (7)	−0.0027 (7)
C6	0.0567 (11)	0.0459 (9)	0.0555 (9)	0.0053 (7)	−0.0059 (8)	−0.0087 (7)
C7	0.0545 (11)	0.0464 (9)	0.0740 (11)	−0.0066 (7)	−0.0105 (8)	−0.0046 (8)
C8	0.0560 (11)	0.0468 (9)	0.0619 (10)	−0.0064 (7)	0.0044 (8)	0.0031 (8)
C9	0.0576 (11)	0.0419 (8)	0.0508 (9)	0.0010 (7)	0.0026 (7)	0.0053 (7)
C11	0.0564 (13)	0.1206 (19)	0.0779 (13)	−0.0055 (12)	0.0030 (10)	−0.0124 (13)
C12	0.0622 (14)	0.1089 (18)	0.0967 (16)	−0.0005 (12)	−0.0056 (12)	0.0081 (14)
C13	0.0641 (15)	0.133 (2)	0.0928 (16)	0.0082 (13)	−0.0021 (12)	−0.0075 (15)
C14	0.0774 (18)	0.138 (2)	0.112 (2)	0.0045 (16)	−0.0112 (15)	0.0073 (18)
C15	0.0728 (18)	0.184 (3)	0.108 (2)	0.0236 (18)	−0.0101 (15)	−0.012 (2)
C16	0.104 (2)	0.209 (4)	0.115 (2)	0.016 (2)	−0.0206 (19)	0.005 (2)
C33	0.0720 (14)	0.0728 (13)	0.0943 (14)	−0.0026 (10)	0.0296 (11)	−0.0245 (12)
C55	0.0834 (16)	0.0945 (16)	0.0711 (12)	0.0065 (12)	0.0193 (11)	−0.0207 (12)

### Geometric parameters (Å, º)

**Table:**

N—C9	1.316 (2)	C11—C12	1.482 (3)
N—C11	1.446 (2)	C11—H11A	0.9700
N—H10	0.8600	C11—H11B	0.9700
O3—C3	1.3703 (19)	C12—C13	1.500 (3)
O3—C33	1.412 (2)	C12—H12A	0.9700
O4—C4	1.3653 (19)	C12—H12B	0.9700
O4—H4	0.8200	C13—C14	1.489 (3)
O5—C5	1.367 (2)	C13—H13A	0.9700
O5—C55	1.412 (2)	C13—H13B	0.9700
O9—C9	1.2373 (19)	C14—C15	1.484 (3)
C1—C6	1.383 (2)	C14—H14A	0.9700
C1—C2	1.386 (2)	C14—H14B	0.9700
C1—C7	1.513 (2)	C15—C16	1.481 (4)
C2—C3	1.384 (2)	C15—H15A	0.9700
C2—H2	0.9300	C15—H15B	0.9700
C3—C4	1.384 (2)	C16—H16A	0.9600
C4—C5	1.392 (2)	C16—H16B	0.9600
C5—C6	1.387 (2)	C16—H16C	0.9600
C6—H6	0.9300	C33—H33A	0.9600
C7—C8	1.522 (2)	C33—H33B	0.9600
C7—H7A	0.9700	C33—H33C	0.9600
C7—H7B	0.9700	C55—H55A	0.9600
C8—C9	1.498 (2)	C55—H55B	0.9600
C8—H8A	0.9700	C55—H55C	0.9600
C8—H8B	0.9700		
			
C9—N—C11	124.19 (16)	H11A—C11—H11B	107.7
C9—N—H10	117.9	C11—C12—C13	113.6 (2)
C11—N—H10	117.9	C11—C12—H12A	108.8
C3—O3—C33	117.97 (14)	C13—C12—H12A	108.8
C4—O4—H4	109.5	C11—C12—H12B	108.8
C5—O5—C55	117.83 (15)	C13—C12—H12B	108.8
C6—C1—C2	119.40 (15)	H12A—C12—H12B	107.7
C6—C1—C7	121.18 (15)	C14—C13—C12	116.4 (2)
C2—C1—C7	119.40 (16)	C14—C13—H13A	108.2
C3—C2—C1	120.40 (16)	C12—C13—H13A	108.2
C3—C2—H2	119.8	C14—C13—H13B	108.2
C1—C2—H2	119.8	C12—C13—H13B	108.2
O3—C3—C4	114.75 (14)	H13A—C13—H13B	107.3
O3—C3—C2	124.57 (16)	C15—C14—C13	115.7 (2)
C4—C3—C2	120.68 (15)	C15—C14—H14A	108.4
O4—C4—C3	122.55 (14)	C13—C14—H14A	108.4
O4—C4—C5	118.68 (15)	C15—C14—H14B	108.4
C3—C4—C5	118.68 (14)	C13—C14—H14B	108.4
O5—C5—C6	124.80 (15)	H14A—C14—H14B	107.4
O5—C5—C4	114.47 (14)	C16—C15—C14	116.1 (3)
C6—C5—C4	120.73 (16)	C16—C15—H15A	108.3
C1—C6—C5	120.10 (15)	C14—C15—H15A	108.3
C1—C6—H6	119.9	C16—C15—H15B	108.3
C5—C6—H6	119.9	C14—C15—H15B	108.3
C1—C7—C8	112.70 (13)	H15A—C15—H15B	107.4
C1—C7—H7A	109.1	C15—C16—H16A	109.5
C8—C7—H7A	109.1	C15—C16—H16B	109.5
C1—C7—H7B	109.1	H16A—C16—H16B	109.5
C8—C7—H7B	109.1	C15—C16—H16C	109.5
H7A—C7—H7B	107.8	H16A—C16—H16C	109.5
C9—C8—C7	112.03 (14)	H16B—C16—H16C	109.5
C9—C8—H8A	109.2	O3—C33—H33A	109.5
C7—C8—H8A	109.2	O3—C33—H33B	109.5
C9—C8—H8B	109.2	H33A—C33—H33B	109.5
C7—C8—H8B	109.2	O3—C33—H33C	109.5
H8A—C8—H8B	107.9	H33A—C33—H33C	109.5
O9—C9—N	121.86 (16)	H33B—C33—H33C	109.5
O9—C9—C8	121.18 (15)	O5—C55—H55A	109.5
N—C9—C8	116.96 (15)	O5—C55—H55B	109.5
N—C11—C12	113.90 (19)	H55A—C55—H55B	109.5
N—C11—H11A	108.8	O5—C55—H55C	109.5
C12—C11—H11A	108.8	H55A—C55—H55C	109.5
N—C11—H11B	108.8	H55B—C55—H55C	109.5
C12—C11—H11B	108.8		
			
C6—C1—C2—C3	−0.4 (2)	C2—C1—C6—C5	0.4 (2)
C7—C1—C2—C3	177.97 (15)	C7—C1—C6—C5	−177.97 (14)
C33—O3—C3—C4	173.79 (16)	O5—C5—C6—C1	−179.81 (14)
C33—O3—C3—C2	−6.2 (3)	C4—C5—C6—C1	0.2 (2)
C1—C2—C3—O3	179.82 (15)	C6—C1—C7—C8	99.72 (18)
C1—C2—C3—C4	−0.2 (2)	C2—C1—C7—C8	−78.67 (19)
O3—C3—C4—O4	−2.5 (2)	C1—C7—C8—C9	−67.73 (19)
C2—C3—C4—O4	177.49 (14)	C11—N—C9—O9	0.6 (3)
O3—C3—C4—C5	−179.18 (14)	C11—N—C9—C8	−179.38 (17)
C2—C3—C4—C5	0.8 (2)	C7—C8—C9—O9	−60.08 (19)
C55—O5—C5—C6	6.1 (2)	C7—C8—C9—N	119.90 (16)
C55—O5—C5—C4	−173.98 (16)	C9—N—C11—C12	−115.0 (2)
O4—C4—C5—O5	2.4 (2)	N—C11—C12—C13	−177.8 (2)
C3—C4—C5—O5	179.19 (14)	C11—C12—C13—C14	177.6 (2)
O4—C4—C5—C6	−177.66 (15)	C12—C13—C14—C15	−177.6 (3)
C3—C4—C5—C6	−0.8 (2)	C13—C14—C15—C16	−179.6 (3)

### Hydrogen-bond geometry (Å, º)

**Table:**

*D*—H···*A*	*D*—H	H···*A*	*D*···*A*	*D*—H···*A*
N—H10···O4^i^	0.86	2.16	2.9655 (19)	155
N—H10···O5^i^	0.86	2.55	3.244 (2)	138
O4—H4···O9^ii^	0.82	1.84	2.6216 (17)	158

Symmetry codes: (i) *x*, −*y*+1/2, *z*+1/2; (ii) *x*, *y*+1, *z*.
